# Characteristics of Presentations to the Emergency Department Following Attempted Suicide with Drugs

**DOI:** 10.3390/ijerph181910232

**Published:** 2021-09-28

**Authors:** Mirjam Kummer, Thomas Müller, Aristomenis K. Exadaktylos, Stephan Krähenbühl, Evangelia Liakoni

**Affiliations:** 1 Clinical Pharmacology and Toxicology, Department of General Internal Medicine, Inselspital, University Hospital Bern, University of Bern, 3010 Bern, Switzerland; mirjam.kummer@insel.ch (M.K.); stephan.kraehenbuehl@usb.ch (S.K.); 2 Translational Research Centre, University Hospital of Psychiatry, 3000 Bern, Switzerland; thomas.mueller@upd.unibe.ch; 3 Privatklinik Meiringen, 3860 Meiringen, Switzerland; 4 Department of Emergency Medicine, Inselspital, University Hospital Bern, University of Bern, 3010 Bern, Switzerland; aristomenis.exadaktylos@insel.ch; 5 Division of Clinical Pharmacology & Toxicology, University Hospital Basel, 4031 Basel, Switzerland

**Keywords:** suicide, suicidal attempt, drug poisoning, emergency department

## Abstract

A relatively high proportion of attempted suicides employ self-poisoning with medication. Data from emergency department presentations can help to identify possible risk drug classes and provide a basis for preventive measures. This retrospective analysis included cases presenting at the emergency department of the University Hospital of Bern, Switzerland, from May 2012 to August 2016, after attempted suicide with drugs. We excluded attempted suicides with only alcohol or other non-medical substances. During the study period, there were 488 cases (466 patients) of attempted suicide with medical substances. The median patient age was 33 years (range 16–93) and 354 (73%) cases were female. The most commonly involved substances/drug classes were benzo-diazepines (*n* = 167, 34%), neuroleptics (*n* = 114, 23%) and paracetamol (*n* = 111, 23%). A total of 231 (47%) cases employed only a single substance. Common symptoms included somnolence (*n* = 245, 50%), tachycardia (*n* = 119, 24%) and nausea/vomiting (*n* = 76, 16%). In most cases, the poisoning was of minor severity (*n* = 231, 47%) and the patients were admitted to a psychiatric hospital (*n* = 264, 54%). Important preventive measures may include careful monitoring for suicidal behaviour when prescribing psychotropic drugs, in addition to restrictions in pack size. Efforts should also be made to enhance the awareness of health professionals qualified to prescribe or supply paracetamol.

## 1. Introduction

Suicide is a major public health concern and suicide prevention a substantial global challenge. Globally, more than 700,000 people die from suicide each year and for each suicide there are many more attempted suicides [1]. Data from 21 countries have been collected from World Mental Health (WMH) surveys of the World Health Organization (WHO) and these have shown that the 12 month prevalence estimate for suicide attempts was 0.3% in developed and 0.4% in developing countries [2], and a history of previous attempted suicide is the most important predictive risk factor for suicide [3]. Among 81 individuals who died by suicide in an observational cohort study, approximately 60% died in the context of the index attempt, and approximately 80% of the 33 who had attempted suicide but did not die in the context of the index attempt, died by suicide within one year [4]. In Switzerland, around 1000 people died by suicide in 2016 (corresponding to a rate of 12/100,000 inhabitants); a 2017 survey reported that 8% of the Swiss population had had thoughts of suicide at least once during the previous two weeks (corresponding to a rate of 7796/100,000 inhabitants) and 0.5% had tried to end their own life within the preceding 12 months (corresponding to 467/100,000 inhabitants) [5]. A previous study from Bern, Switzerland, investigated patients presenting at the emergency department (ED) due to poisoning between 2004 and 2007 and found that, attempted suicide was the most common cause of severe cases in women and one-fifth of the cases in men [6].

According to reports based on hospital data on suicide patterns in the Bern agglomeration from 2004 to 2010, drugs were most commonly used in attempted suicide, most frequently analgesics, tranquilisers/hypnotics, neuroleptics and antidepressants [7]. Also according to data from presentations to medical institutions in Basel, Switzerland, a relatively high proportion of attempted suicides accounts for self-poisoning with drugs, in particular nonsteroidal anti-inflammatory drugs (NSAID), benzodiazepines and antidepressants [8], whereas antidepressants, neuroleptics and benzodiazepines were the most commonly used single substances in cases admitted to the intensive care after a suicide attempt [9]. Data from emergency department presentations are also available from other countries. For example, analgesics/antipyretics/antirheumatics, sedatives/hypnotics/tranquilisers, and other psychotropic agents have been found to be involved in about one-half of presentations with self-inflicted poisoning in the United States [10]. In the Republic of Ireland and in Northern Ireland, tranquilisers were most commonly involved in intentional drug overdoses [11]. In Belgium, antidepressants and sedatives/tranquilisers were used in 75% of cases of self-poisoning by drug overdose (the method used in 81% of the self-harm presentations) [12]. In Korea, the most commonly used drugs were sedatives/hypnotics (most commonly “Z-drugs”, a class of drugs structurally different to benzodiazepines but with a similar mechanism of action, and the benzodiazepine alprazolam) and analgesics [13].

In England, data on self-harm have been recorded in recent decades within the framework of the Multicentre Study of Self-harm, which contributes to the National Suicide Prevention Strategy [14]. Recently published data from this project for the period 2004 to 2014 indicate that approximately one-third of presentations to the ED for intentional drug overdose were associated with paracetamol monointoxications [15]. In Switzerland, data on suicide attempts were previously collected in the framework of the WHO/EURO Multicenter Study on Monitoring Suicidal Behaviour in Europe (MONSUE) at two locations between 2004 and 2010 (Basel 2003–2006 [8], Bern 2004–2010 [7]). However, with the exception of some data collected in recent years under the direction of the Groupe Romand Prévention Suicide (GRPS) at some EDs in the French part of Switzerland [16] and self-reported data from surveys [5], such data are currently not routinely collected in Switzerland [17].

Trends in the drugs used in attempted suicide might vary not only between countries, but also over time, reflecting changes in prescribing practice and drug availability [12], in addition to between age groups [18], which possibly also represents differences in prescription patterns. Other and less expected factors may also affect such trends, as demonstrated for example by an increase in hospital presentations due to self-poisoning in the United Kingdom after depiction of paracetamol self-poisoning in an episode of a popular television drama [19].

Bearing in mind that, in contrast to suicides, attempted suicides are currently not routinely recorded in Switzerland [17], data collected from ED presentations can provide important information on attempted suicides with medication and thus help to identify risk drug classes and preventive measures. Because local differences have been identified in previous reports and there are currently only sparse recent data available from studies investigating these aspects and trends in Switzerland on a large patient population, the present study aimed to describe ED presentations following attempted suicide with drugs at a large urban ED in Bern, Switzerland.

## 2. Materials and Methods

This was a retrospective single centre study at the ED of the University Hospital of Bern, Switzerland, from 10 May 2012 to 31 August 2016. The ED of the University Hospital of Bern serves both as a primary care facility (walk-in patients) and a tertiary referral centre for other hospitals in the greater Bern area, with about 48,000 emergency admissions per year (2018). The study was approved by the local ethics committee (No. 2016-01850) and included patients ≥16 years of age presenting to the ED after attempted suicide with drugs.

Cases were identified using a search function of the electronic ED patient database E.care. This electronic database stores all patient data collected during routine clinical care at the ED and allows recall of past diagnostic reports, consultations, and other relevant medical documents. The database was searched using appropriate full-text search terms (e.g., “suicidal attempt”, “suicide”, “suicidal”). Each identified case was reviewed by one of the authors of the study. A drug was defined as a compound that is taken as approved/registered medical product in the context of a medical indication and included prescription and “over the counter” (OTC) medication. The drug(s) associated with the attempted suicide were identified on the basis of the patient’s self-report, or information retrieved from witnesses. Attempted suicide was defined as a self-injurious behaviour that was intended to end one’s life but that was non-fatal [20,21]. Cases were excluded if the patient had left the ED before being seen by the ED staff; also excluded were cases with unintentional intoxication or intoxication in the context of a medical indication or recreational use without suicidal intention, patients attending the ED for a follow-up that was not in the context of acute attempted suicide, and attempted suicides involving only alcohol or other non-medical substances and chemicals.

The following data were exported (if available) from E.care for the analyses: sex, age, type of transport to the ED (e.g., by ambulance), day and time of presentation, drug(s) involved, concomitant useof alcohol or recreational drugs, clinical symptoms and variables, severity of poisoning, and outcome. Night arrival refers to ED presentation between 10 p.m. and 7 a.m., and weekend arrival to between Saturday 7 a.m. and Sunday 10 p.m. Clinical variables included the Glasgow Coma Scale (GCS) score, heart rate, blood pressure, respiratory rate, body temperature, laboratory tests and electrocardiography (ECG) findings. Hyperthermia was defined as a temperature ≥39 °C, measured by any method, hypothermia as a temperature <35 °C, hypertension as systolic blood pressure ≥180 mmHg, hypotension as systolic blood pressure ≤90 mmHg, tachycardia as a heart rate of >100 beats per minute (bpm), bradycardia as a heart rate of <60 bpm, tachypnoea as a respiratory rate of >20 breaths per minute, and bradypnoea as a respiratory rate of <10 breaths per minute. Respiratory depression includes cases in which periods of apnoea or hypoventilation were documented by the medical staff before or during the presentation.

In terms of laboratory outcome, drug-induced liver injury (DILI) was defined as an elevation of alanine aminotransferase (ALT) over 5× the upper limit of normal (ULN), or elevation of alkaline phosphatase (ALP) over 2× ULN, or elevation of ALT over 3× ULN and simultaneous elevation of bilirubin over 2× ULN [22]. Impaired renal function was defined as an estimated glomerular filtration rate (eGFR) under 60 mL/min according to internal laboratory standard values. Creatine kinase (CK) elevation as a marker for muscle injury was registered above a specified level of 190 U/L for men and 170 U/L for women.

A QTc interval prolongation was defined as a QTc >470 ms in men and QTc >480 ms in women [23]. Results of toxicological drug screening were available when required by the attending emergency physician and were based on a urinary drug immunoassay (Triage^®^ TOX Drug Screen, Alere Inc, Cologne, Germany) for amphetamines, barbiturates, benzodiazepines, cocaine, methadone, methamphetamines (including MDMA), opiates, phencyclidine, tricyclic antidepressants, paracetamol and tetrahydrocannabinol (THC) [24]. The severity of poisoning was assessed using the “Poisoning Severity Score” for the grading of acute poisoning [25].

Data were analysed descriptively using Microsoft Excel software. Numerical data are presented as arithmetic means and standard deviations (±SD) or medians and range, and nominal data as proportions (%).

## 3. Results

Over the study period, there were 169,175 ED attendances in total. Of these, 3012 potential cases were initially retrieved using the full-text search terms—when at least one of the terms was mentioned in at least one of the fields of the ED report (including cases with the terms not mentioned in relation to the current presentation, in addition to documentation of, e.g., “suicidal intention denied” or similar). Of these, 920 were cases with ED presentation related to attempted suicides, with 578 among those related to intake of substances. Of these, 73 had to be excluded because of no intentional intake (unintentional overdose, medical error) or recreational use, and 17 because they involved non-medical chemicals. Finally, 488 cases were included in the analysis, corresponding to 466 patients (12 patients presented twice, one patient three times, one patient four times and one patient six times during the study period).

The mean (±SD) age of the study population was 37 ± 16 years (median 33, range 16–93) and 354 (73%) cases were female. Further characteristics of the included cases are shown in Table 1.

**Figure 1:**
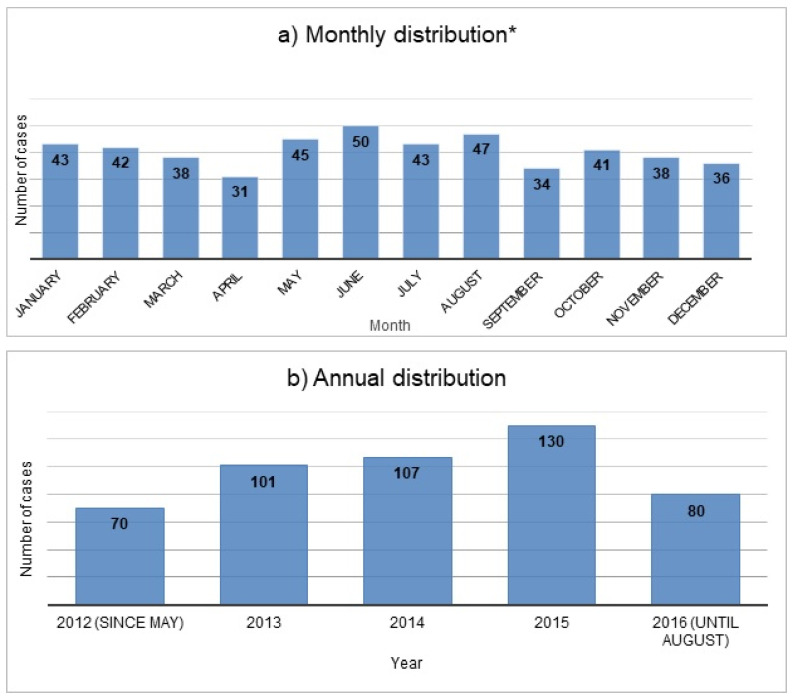
Monthly * (**a**) and annual (**b**) distribution of cases presenting due to self-poisoning from drugs during the study period (N = 488). * Due to the time period of the study, the months May, June, July and August are represented for one year longer than for the other months.

**Figure 2:**
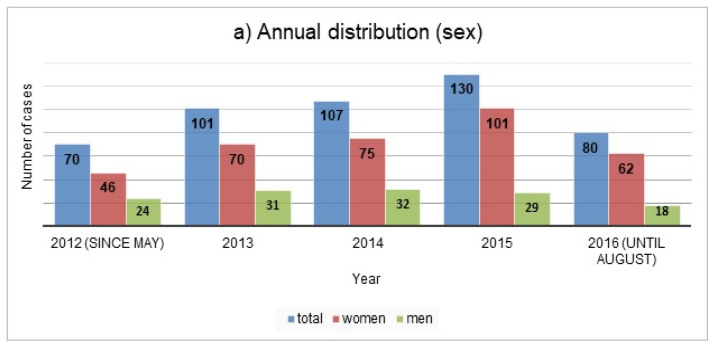

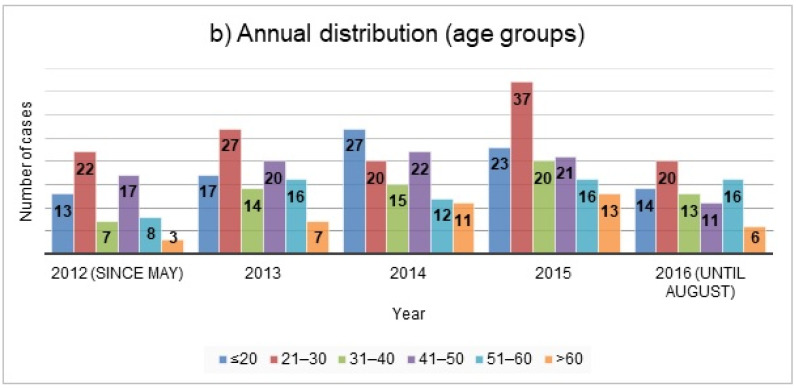
Annual distribution of the cases presenting due to self-poisoning from drugs by sex (**a**) and by age groups (**b**) (*N* = 488).

Figure 1 shows the annual and monthly distribution of the cases during the study period; the annual distribution is further illustrated by sex and age in Figure 2.

**Figure 3:**
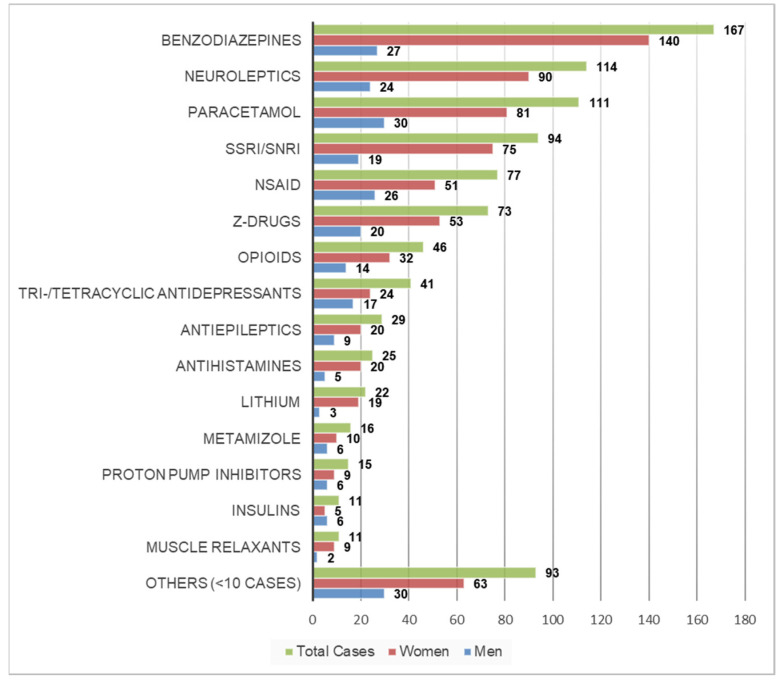
Most commonly reported substances/drug classes in cases presenting due to attempted suicide with drugs as total number of cases and by sex (*N* = 488; more than one drug involved in some cases). NSAID: non-steroidal anti-inflammatory drugs; SSRI: selective serotonin reuptake inhibitors; SNRI: serotonin-norepinephrine reuptake inhibitors.

Table 2 shows the reported drugs used in the attempted suicides; substances/drug classes involved in more than 10 cases are also shown in Figure 3 as total number of cases and by sex.

Among the total cases and also among women, benzodiazepines were the most commonly reported drugs (*n* = 167 and *n* = 140, respectively), followed by neuroleptics (*n* = 114 and *n* = 90, respectively) and paracetamol (*n* = 111 and *n* = 81, respectively); among men the most commonly reported substances were paracetamol (*n* = 30), followed by benzodiazepines (*n* = 27) and NSAID (*n* = 26) (Figure 3). Sedatives (i.e., benzodiazepines and/or Z-drugs) were involved in 240 cases (49%), non-opioid analgesics (i.e., paracetamol, NSAID and/or metamizole) in 204 cases (42%) and antidepressants (i.e., selective serotonin reuptake inhibitors (SSRI), serotonin-norepinephrine reuptake inhibitors (SNRI) and tri- and/or tetracyclic antidepressants) in 135 cases (28%).

**Figure 4:**
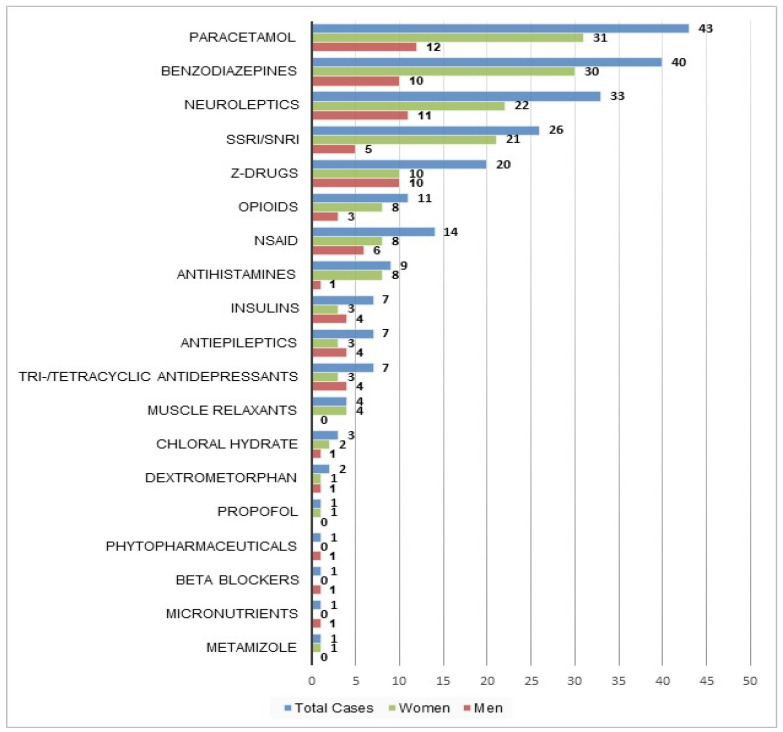
Substances involved in monointoxication cases as total number of cases and by sex (*n* = 231).

In 231 cases (47%), only one substance was used in the attempted suicide. The most common substances involved in these cases were similar to those for total cases (Table 2 and Figure 4).

Among monointoxications, sedatives (i.e., benzodiazepines and/or Z-drugs) were involved in 60 cases (26%), non-opioid analgesics (i.e., paracetamol, NSAID and/or metamizole) in 58 cases (25%) and antidepressants (i.e., SSRI, SNRI, tri- and/or tetracyclic antidepressants) in 33 cases (14%).

**Figure 5:**
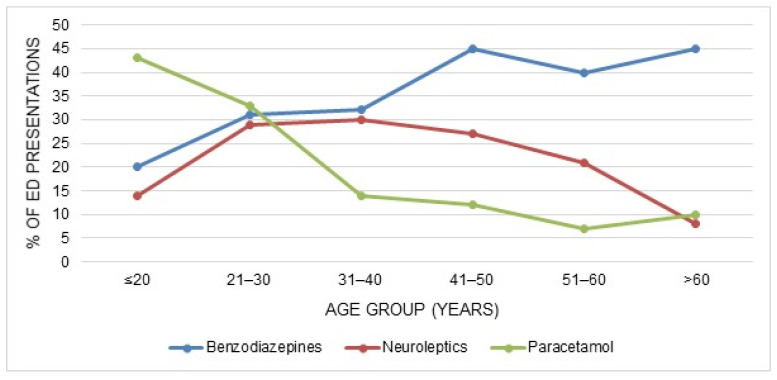
Age distribution of the three most commonly reported substances/drug classes in cases presenting due to attempted suicides with drugs.

The age distribution of the three most commonly reported drugs/drug classes is shown in Figure 5; the proportion of the age groups presenting due to self-poisoning with drugs varied among the different substances/drug classes. 

**Figure 6:**
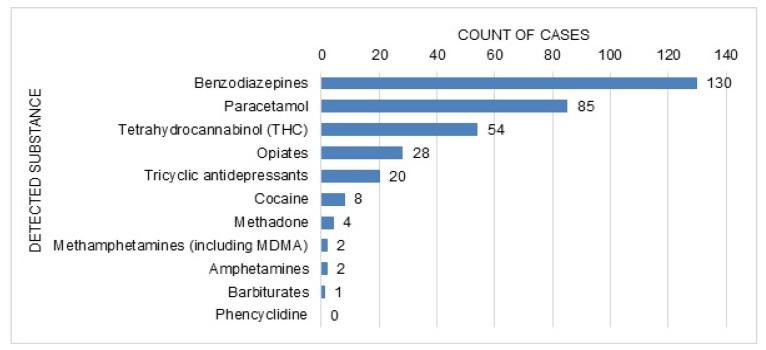
Analytically detected substances in cases with available urine immunoassay test (*n* = 306).

Twenty patients reported concomitant recreational drug use (heroin in eight, cannabis in seven and cocaine in five cases, followed by amphetamines, methadone and opioids/opiates in one case each). Three of those patients reported the use of more than one substance. A toxicological urine drug screening test was performed in 306 (63%) cases, with a positive result in 216 cases. The detected substances are shown in Figure 6. In 132 of the positive cases, only one substance was found in the urine screening; in 84 cases two or more substances were detected (the most common combination was benzodiazepines with THC, in 25 cases). The test was negative in 90 cases, and in 182 cases no toxicological screening test was performed.

Table 3 summarises the clinical symptoms and/or signs and laboratory findings of the patients seen by medical and paramedical staff or reported by the patient or witnesses.

In addition to the clinical and laboratory findings, electrocardiography findings were documented in the reports in 336 (69%) cases. Among these, 93 cases of dysrhythmias (four arrhythmic, 72 of tachycardia, 17 of bradycardia) were detected. Twenty-seven patients (24 female and three male) showed a prolonged QTc interval.

In most cases, the intoxication was of minor severity (Table 4).

## 4. Discussion

This retrospective study describes presentations at a large urban ED in Bern, Switzerland, following attempted suicide with drugs. Over a period of nearly four and a half years (May 2012–August 2016), there were approximately nine presentations/month due to drug intoxications with suicidal intent. The typical patient was female, relatively young (16–30 years old), had ingested more than one substance, and presented with somnolence, tachycardia or nausea. Apart from paracetamol, the vast majority of presentations were related to prescription drugs used in the treatment of psychiatric disorders and sedatives were the largest group of substances. Concomitant use of alcohol was involved in a little less than one-third of the cases and was clearly more common than concomitant use of recreational drugs. In terms of outcome, most presentations were of minor severity and were treated in a psychiatric hospital after the stay in the ED.

Women were highly represented in our study, which is consistent with previous data from Switzerland, according to which suicide attempts were more common in women than in men [7,8]; poisoning was the most common method of suicide in women, whereas men more often choose more lethal methods, such as firearms or hanging [26]. Studies from other European countries and from Korea investigating presentations after deliberate self-poisoning with medication have similarly found that most patients were female [12,13,18,27]. Female sex was also among the identified risk factors for suicidal behaviour in data from a large cross-national epidemiological survey of the WHO [2]. In contrast to attempted suicides, suicide rates are higher in men; however, this is more prominent in high income countries (male-to-female ratio 3.5 in high vs. 1.6 in low- and middle-income countries) [3].

We found that paracetamol and sedatives were the two most common substances/drug groups used in attempted suicide with drugs. This is consistent with an earlier study from the Bern agglomeration for the period between 2004 and 2010, which found that frequently used substance groups were analgesics, tranquilisers and hypnotics [7]. Neuroleptics were also among the most commonly used substance group in this previous study [7], and the third most frequently used substance group in the present study; this may be due to the increasing off-label use of these substances (e.g., quetiapine) as tranquilisers or anxiolytics [28,29]. Our observed substance distribution was also similar to a previous study from Basel [8], another city in Switzerland with a large urban ED, where benzodiazepines and other sedatives were most commonly reported, followed by neuroleptics and NSAID (paracetamol was not mentioned separately in this analysis). In contrast, in other countries such as the United States, in line with the local opioid epidemic, a relatively high proportion of attempted suicides from self-poisoning is associated with opiates and other related narcoleptics [30,31]; in the present study these substances were involved in only about 10% of the cases.

In more than half the cases, more than one substance was ingested, which could make it more difficult for physicians to choose the adequate treatment, because the clinical consequences of mixed intoxications are harder to anticipate. Furthermore, substance patterns differed between the age groups: Whereas patients under 30 years most commonly used paracetamol, psychotropic drugs were the most common drug class in older patients. These variations by age are consistent with findings of studies in the United Kingdom and Ireland, where paracetamol was most commonly involved in intentional drug overdoses in younger age groups, and the proportion of patients using psychotropic drugs generally increased with age [11,18,27,32]. This may reflect differences in prescribing patterns and drug availability: older patients are more likely to be receiving treatment for psychiatric disorders, and thus have access to psychotropic medication. In addition, in most countries, including Switzerland, paracetamol can be bought as an OTC drug (the maximal paracetamol OTC pack contains 10 g) [33]. It is therefore readily available for younger individuals without access to prescribed medication.

In our population, concomitant alcohol use was common and more frequent than described in the previous study from Bern on attempted suicides, whereby in this previous study, the proportion of alcohol involvement was reported for all methods of suicide attempts [7]. If we compare our findings to international data, alcohol was more rarely involved in our study population than, e.g., in Ireland [11,32], suggesting there might be national differences in patterns of alcohol abuse [11,34]. It should be considered that concomitant alcohol use may have led to an overrepresentation of neurological and gastrointestinal symptoms, such as somnolence and nausea/vomiting, and that impaired consciousness may be associated with a higher percentage of severe intoxications in our study. In contrast to concomitant alcohol ingestion, concomitant use of recreational drugs was reported only by a minority of the patients. The greater number of positive results for urinary toxicological drug screening may be due to underreporting of (illegal) drugs, substances that can be found in urine beyond the time period of acute intoxication (e.g., THC), or due to further limitations of the urine immunoassay such as false positive results (e.g., cross reactivity with other compounds).

Our findings have important implications for public health. Because our results show that the majority of patients presenting after attempted suicide with drugs belong to the younger age group in which paracetamol was the commonest drug used, measures such as pack size restrictions should be considered. Several studies from the UK have demonstrated a link between the availability of paracetamol and the frequency of its use for suicide. Legislation leading to a reduction in the OTC pack sizes had a beneficial impact on intentional overdoses and related hospital admissions [35,36]. However, in contrast to these findings, other UK data have shown that regulations restricting the pack size did not clearly reduce paracetamol-related deaths [37], and a systematic review reported that although these regulations may have led to reduced hospital and liver unit admissions, there were several limitations to the included studies, so that no final conclusion could be drawn [38]. When trying to find appropriate preventive measures, it should also be considered that suicide rates are greater in older individuals, even though the rates of attempted suicide are lower compared to younger adults [39,40]; this highlights the importance of preventive measures such as adequate treatment of chronic pain and other medical conditions, and early identification of red flags such as recent loss of a spouse, depression and social isolation in older patients [39]. Another important finding of our study is that sedative-hypnotics and neuroleptics were the most commonly used substances. Benzodiazepines, Z-drugs and neuroleptics are widely prescribed in Switzerland [29], not only by psychiatrists, but also by general practitioners. On the basis of our results, experience of suicidal thoughts should be regularly evaluated when prescribing these drugs to patients, especially when pre-existing psychiatric disorders are known. Therefore, additional preventive measures might include special training for primary care physicians or restricting long-term prescription of these drugs to specialists. In patients with a history of attempted suicide, psychosocial therapy can contribute to a reduction in the risk of a subsequent attempt and mortality [41].

The limitations of our study include the retrospective design and the lack of standardised data collection at the ED. In addition to missing data, we cannot exclude the possibility that some cases were missed, if the ingestion of drugs was unknown to the medical staff at the moment of the ED presentation due to, e.g., impaired consciousness. Moreover, a single centre study with data from one ED may not be representative for the whole country and we did not have access to data about long-term outcomes when the patients were transferred to an external hospital after the ED presentation. In addition, no data about the presence of psychiatric comorbidities or previous suicide attempts were analysed and information about previous laboratory results (e.g., pre-existing renal insufficiency) was not included. Furthermore, in cases with ingestion of multiple drugs, it is unclear which substance was mainly responsible for the clinical manifestations. The strengths of the study include the sensitive search and the individual review of each case. To our knowledge, this is currently the only study in Switzerland in the past decade that describes case characteristics and substance patterns of drug self-poisoning in ED presentations in a larger patient population. Therefore, it may contribute to identifying current risk drug classes and susceptible groups for guiding future targeted public health measures.

## 5. Conclusions

Most of the substances used for self-poisoning in our study were prescription drugs used in the treatment of psychiatric disorders. Therefore, when prescribing psychotropic drugs, periodic monitoring of patients for suicidal thoughts may be a crucial preventive measure. Furthermore, processes for monitoring self-harm behaviour should be organised and optimised (where already available). This may also contribute to preventive strategies, especially in the light of the increased risk of suicide after a prior attempt [3]. Moreover, measures to raise awareness among health care providers prescribing or dispensing paracetamol should be considered, because this substance is readily available in Switzerland and is predominantly used for attempted suicide with drugs in younger age groups.

## Figures and Tables

**Table 1:** Main characteristics of cases presenting due to attempted suicide with drugs (*N* = 488).

	Number of Cases (%)
**Sex**	
Male	134 (27)
Female	354 (73)
**Age group (years)**	
≤20	94 (19)
21–30	126 (26)
31–40	69 (14)
41–50	91 (19)
51–60	68 (14)
>60	40 (8)
**Time of Presentation**	
Night arrival	141 (29)
Weekend arrival	144 (30)
**Admission to ED**	
Self-presentation	84 (17)
Brought by rescue services/police	404 (83)
**Reported Concomitant Use**	
Ethanol	138 (28)
Recreational drug use	20 (4)
**Consumption of multiple drugs**	
Yes	258 (53)
No	230 (47)

**Table 2:** Reported substances/drug classes (count of cases) involved in presentations due to attempted suicides with drugs (total cases, *N* = 488; monointoxication cases, *N* = 231; more than one drug involved in some cases).

Substance/Drug Class	Number of Total Cases (% of Total Cases)	Number of Monointoxication Cases (% of Monointoxication Cases)
Benzodiazepines	167 (34)	40 (17)
Neuroleptics	114 (23)	33 (14)
Paracetamol	111 (23)	43 (19)
SSRI/SNRI	94 (19)	26 (11)
NSAID	77 (16)	14 (6)
Z-Drugs	72 (15)	20 (9)
Opioids	46 (9)	11 (5)
Tri-/Tetracyclic antidepressants	41 (8)	7 (3)
Antiepileptics	28 (6)	7 (3)
Antihistamines	25 (5)	9 (4)
Lithium	22 (5)	
Metamizole	16 (3)	1 (<1)
PPI	15 (3)	
Muscle relaxants	11 (2)	4 (2)
Insulins	11 (2)	7 (3)
Micronutrients	8 (2)	1 (<1)
ACE inhibitors	7 (1)	
Diuretics	7 (1)	
Antispasmotics	7 (1)	
Beta blockers	6 (1)	1 (<1)
Lipid reducers/statins	6 (1)	
Dextrometorphan	5 (1)	2 (1)
Phytopharmaceuticals	5 (1)	1 (<1)
Calcium antagonists	4 (1)	
Sympathomimetics	4 (1)	
Chloral hydrate	3 (1)	3 (1)
Methylphenidate	3 (1)	
Thyroid hormones	3 (1)	
Antibiotics	3 (1)	
Anticoagulants	2 (<1)	
Agomelatine	2 (<1)	
Levodopa	2 (<1)	
Antiemetics	2 (<1)	
Loperamide	2 (<1)	
Propofol	2 (<1)	1 (<1)
Metformin	2 (<1)	
Allopurinol	2 (<1)	
Laxatives	1 (<1)	
Mycophenolate	1 (<1)	
Digoxin	1 (<1)	
Nicorandil	1 (<1)	
Erdosteine	1 (<1)	
Estrogen	1 (<1)	

ACE: angiotensin converting enzyme; NSAID: non-steroidal anti-inflammatory drugs; PPI: proton pump inhibitors; SSRI: selective serotonin reuptake inhibitors; SNRI: serotonin-norepinephrine reuptake inhibitors.

**Table 3:** Clinical features of the cases presenting due to attempted suicide with drugs during the study period (*N* = 488).

	Number of Cases (%)
***On arrival***	
Mildly impaired consciousness, GCS 13–14	115 (24)
Moderately impaired consciousness, GCS 9–12	33 (7)
Unconscious, GCS <9	21 (4)
Tachycardia	95 (19)
Tachypnoea	49 (10)
Bradycardia	13 (3)
Hypertension	9 (2)
Hypotension	8 (2)
Hypothermia	6 (1)
***Before or during the presentation***	
**Cardiovascular symptoms**	
Tachycardia	119 (24)
Hypotension	33 (7)
Hypertension	28 (6)
Bradycardia	12 (2)
Arrhythmias	1 (<0.5)
Cardiovascular arrest	1 (<0.5)
**Respiratory symptoms**	
Tachypnoea	51 (10)
Hypoxia	13 (3)
Bradypnoea	8 (2)
Respiratory depression	8 (2)
Dyspnea	5 (1)
**Neurological symptoms**	
Somnolence	245 (50)
Unconsciousness (defined as lowest GCS <9)	50 (10)
Agitation	26 (5)
Confusion/Desorientation	20 (4)
Vertigo	19 (4)
Cephalgia	13 (3)
Dysarthria	13 (3)
Seizures	10 (2)
Mydriasis	7 (1)
Nystagmus	5 (1)
Miosis	4 (1)
Myoclonia	4 (1)
Extrapyramidal symptoms	4 (1)
Ataxia	3 (1)
Tremor	3 (1)
Hallucinations	2 (<0.5)
Paresthaesia	2 (<0.5)
Amnesia	1 (<0.5)
**Gastrointestinal symptoms**	
Nausea or vomiting	76 (16)
Abdominal pain	26 (5)
Diarrhoea	3 (1)
**Laboratory findings**	
Elevated creatine kinase (CK)	35 (7)
CK >5 ULN	4 (1)
Impaired renal function (eGFR <59 mL/min/1.73 m^2^)	21 (4)
Drug Induced Liver Injury (DILI)	6 (1)
**Miscellanous**	
Hypothermia	9 (2)
Hypoglycemia	5 (1)
Dry mouth	3 (1)
(Lactic-)acidosis	3 (1)
Sweating	3 (1)
Urinary retention	2 (<0.5)
Tinnitus	1 (<0.5)
Coagulation disorder	1 (<0.5)
Hyperthermia	1 (<0.5)

**Table 4:** Severity of poisoning and outcome data in the cases presenting due to attempted suicide with drugs during the study period (*N* = 488).

	Number of Cases (%)
**Poisoning Severity Score**	
None	71 (15)
Minor	231 (47)
Moderate	131 (27)
Severe	55 (11)
**Outcome**	
Discharged	61 (13)
Hospitalisation (ICU)	100 (20)
Hospitalisation (normal ward or IMC)	58 (12)
Transfer to psychiatric ward/external psychiatric hospital	264 (54)
Transfer to external hospital	5 (1)

ICU: intensive care unit; IMC: intermediate care unit.

## Data Availability

Not publicly available, data supporting the findings of the study are available within the article.
